# Cigarette smoking and its association with serum lipid/lipoprotein among Chinese nonagenarians/centenarians

**DOI:** 10.1186/1476-511X-11-94

**Published:** 2012-07-24

**Authors:** Zhang Yan-Ling, Zhao Dong-Qing, Huang Chang-Quan, Dong Bi-Rong

**Affiliations:** 1 Department of Geriatrics, West China Hospital, Sichuan University, Guoxuexiang 37, Chengdu, Sichuang province, China, 610041

**Keywords:** Serum lipid/lipoprotein, Cigarette smoking, Nonagenarians/Centenarians

## Abstract

**Objective:**

Cigarette smoking had been confirmed as an increased risk for dyslipidemia, but none of the evidence was from long-lived population. In present study, we detected relationship between cigarette smoking habits and serum lipid/lipoprotein (serum Triglyceride (TG), Total cholesterol (TC), Low-density lipoprotein (LDL) and high-density lipoprotein (HDL)) among Chinese Nonagenarians/Centenarian.

**Methods:**

The present study analyzed data from the survey that was conducted on all residents aged 90 years or more in a district, there were 2,311,709 inhabitants in 2005. Unpaired Student’s *t* test, *χ*^2^ test, and multiple logistic regression were used to analyze datas.

**Results:**

The individuals included in the statistical analysis were 216 men and 445 women. Current smokers had lower level of TC (4.05 ± 0.81 vs. 4.21 ± 0.87, *t* = 2.403, P = 0.017) and lower prevalence of hypercholesteremia (9.62% vs. 15.13%, *χ*^2^ = 3.018,P = 0.049) than nonsmokers. Unadjusted and adjusted multiple logistic regressions showed that cigarette smoking was not associated with risk for abnormal serum lipid/lipoprotein.

**Conclusions:**

In summary, we found that among Chinese nonagenarians/centenarians, cigarette smoking habits were not associated with increased risk for dyslipidemia, which was different from the association of smoking habits with dyslipidemia in general population.

## Introduction

Dyslipidemia was the presence of abnormal levels of lipids in the blood, characterized by an elevation of the concentration of total cholesterol (TC), low-density lipoprotein (LDL), and triglycerides (TG), and a decrease in high-density lipoprotein cholesterol (HDL) [1,2]. Smoking was a major risk factor for atherosclerotic cerebro- and cardiovascular diseases (CVD) through leading to dyslipidemia [3]. A comprehensive meta-analysis by Craig et al. examined published data from 1966 to 1987 and estimated the excess risk posed by smoking on CVD, with particular emphasis on lipid and lipoprotein involvement [1]. Results of their analysis indicated that compared with non-smokers, cigarettes smokers had significantly higher TC (3%), TG (9.1%), and LDL (10.4%), higher (but not significant LDL (1.7%), and lower concentrations of HDL (−5.7%) [1]. In the meta-analysis, a dose–response relationship was found between the number of cigarettes smoked and the change in lipid or lipoprotein variable [1]. The results indicated a progressive increase (%) as the smoking dosage increased from none to heavy: TC (0, +0.8, +4.3 and +4.5%), TG (0, +10.7, +11.5 and +18.0%), LDL (0, +7.2, +44.4, and +39.0%), and LDL (0, −1.1, −1.4 and +11.0%). They also reported dose related decreases in HDL (0, −4.6, −6.3, −8.9%) [1].

Many physiologic benefits were associated with smoking cessation, including normalization of the lipid and lipoprotein profile. A meta-analysis by Maeda et al. suggested that with smoking cessation an individual could experience an increase in HDL, but other lipid and lipoproteins (TC, LDL, and TG) remained unchanged [4]. Movement toward normalization of HDL could be seen in as little as 17 days and would continue to progress toward normal (non-smoking) levels as long as cessation continues. These results had important implications, because they would alter the ratios of HDL, TC, HDL and LDL and could promote clearance of cholesterol from the circulation [4].

The relationship between dyslipidemia and smoking, smoking dosage or smoking cessation had been confirmed by sufficient evidence [5-8]. There was a high smoking rates in China, especially in elderly men, and several millions old people die from smoking-related diseases and dyslipidemia-related diseases in china. Smoking and dyslipidemia synergy to accelerate the death, there might be a difference relationship between smoking and dyslipidemia, clearing the relationship might be important for increasing the proportion of the longevity in population and for improving the health status of the longevity of the elderly. However, all the evidences on association of dyslipidemia with smoking habits were from general population. So it still had been unclear that whether the relationship existed in long-lived subjects or not. In the present study, using data from a sample of Chinese nonagenarians and centenarians, we examined the association between hypertension and dyslipidemia in the long-lived subjects.

## Subjects and methods

### Study subjects

The methods were reported previously [9-11]. In brief, on the basis of the Dujiangyan (located in Sichuan province, southwest China) 2005 census, a cross-sectional study for age-related diseases was conducted in 870 long-lived subjects (>90 years), which was a part of the Project of Longevity and Aging in Doujiangyan (PLAD). The PLAD aimed to investigate the relationship between environments, lifestyle, genetic, longevity and age-related diseases. Volunteers were examined by trained professional physicians according to basic health criteria. And the results were filled in the standard form. In this analysis, the subjects, who had no biological specimen for measurement levels serum Lipid/Lipoprotein, or reported the use of drugs affecting the serum lipid/lipoprotein levels, or no information on smoking habits were excluded. The study population ultimately consisted of 661 long-lived subjects. Informed consents were obtained from all participants (as well as their legal proxies). The Research Ethics Committee of the Sichuan University approved the study.

## Data collection and measurements

### Measurement of serum lipid/lipoprotein and fasting blood glucose (FBG)

The methods were reported previously [10,11]. Blood samples were collected after an overnight fast (at least 8 h) for measurement of serum lipid/lipoprotein. Lipid/lipoprotein levels including serum Triglyceride (TG), TC, Low-density lipoprotein (LDL) and High-density lipoprotein (HDL) were determined by standard laboratory techniques. Cholesterol and triglyceride levels were measured by commercially available enzymatic kits (Roche, Mannheim, Germany) [12,13]. FBG was also determined by the standard laboratory techniques [12,13]. FBG was assessed using a glucose oxidase assay [14]. Lipid/lipoprotein levels and FBG was performed by a technician in the biochemistry laboratory of Sichuan University. Abnormal levels of serum lipid/lipoprotein were defined according to the criteria provided by the Chinese Medical Association 2004. Abnormal criteria were: TC >5.18 mmol/l, TG >1.7 mmol/l, LDL >3.37 mmol/l and HDL <1.04 mmol/l [15].

## Cigarette smoking

Information on smoking habits was inquired using the questionnaire designed according to the previous studies [8,16-18]. Participants was classified the following groups according to the questionnaire [19,20]: 1) nonsmokers: individuals who reported never having a cigarette; 2) smokers: individuals who reported smoking every day now and having the habit for more than 6 months; for smokers, the smoking dose was questioned: how many cigarette smoking was taken and the smokers were grouped as follows: light (1–4 cigarette per day), medium (5–9 cigarette per day), heavy (above 10 cigarette per day). 3) With a history of smoking: individuals who had smoking for at least 6 months and including those smoking former and current; 4) smoking cessation: individuals who had quit smoking for at least 6 months; Participants who smoked intermittently (0 or 1 cigarette per day) were not included in the study.

## Statistical analysis

All of the statistical analyses for this study were performed with the SPSS for Windows software package, version11.5 (SPSS Inc, Chicago, Illinois, USA). Serum lipid/lipoprotein levels and prevalence of abnormal levels of serum lipid/lipoprotein were compared between smokers and nonsmokers, or among groups with different smoking dosage, or with and without history of smoking, or with and without smoking cessation using *χ*^2^ or Fisher’s exact test (where an expected cell count was < 5) for categorical variables and unpaired Student’s *t* test for continuous variables. Multiple logistic regression was used to estimate the odds ratio (OR) and 95% confidence interval (CI) of current smoking and smoking cessation as a function of increased abnormal levels of serum lipid/lipoprotein. P value <0.05 was considered to be statistically significant, and all of the P values have two sides.

## Results

### Prevalence of abnormal serum lipid/lipoprotein and smoking habits

Among the 661 participants, mean age was 93.51 years, 69 were centenarians and 445 were women. Ninety percent of participants lived in the country side. There were 401 individuals with history of smoking (60.67%), including 128 (19.36%) individuals with smoking cessation and 273 (41.30%) current smokers. The mean of FBG was 4.64 (s.d.:1.45). In those current smokers, 146 (53.48%), 90 (32.97%) and (13.55%) were in the light, medium and heavy dosage groups, respectively. Among the sample, men had more current smokers (64.16% % vs. 15.96%, *χ*^2^ = 87.88, P ≤ 0.001) and more with history of smoking (78.76% vs. 50.11%, *χ*^2^ = 63.12, P ≤ 0.001) than women.

Among the sample, the means of serum Lipid/Lipoprotein were 4.15 (s.d.:0.85) mmol/L, 1.22 (s.d.:0.64) mmol/L, 2.28 (s.d.:0.97) mmol/L, 1.58 (s.d.:0.59) mmol/L for TC, TG, LDL and HDL, respectively. The prevalence of abnormal serum Lipid/Lipoprotein were 75 (11.3%), 89 (13.5%), 35 (5.3%) and 37 (5.6%) according to TC, TG, LDL and HDL, respectively. (See Tables 1 and 2).

**Table 1:** Comparing the age, gender, FBG and serum lipid/lipoprotein between subjects with smoking and non-smoking habit, and among groups with different smoking quantity (n = 661)

**Characteristics**	**Smoking status**				**Smoking dosage**				
	**Smoker n = 273**	**Non-smoker n = 388**	***χ*2 or*t***	**P value**	**Light n = 146**	**Medium n = 90**	**Heavy n = 37**	***χ*2 or*F***	**P value**
Age (years)	93.37 ± 3.22	93.62 ± 3.47	0.944	0.346	93.52 ± 3.20	93.23 ± 3.13	93.11 ± 3.56	0.362	0.697
Gender ^a^									
(Male/Female)	145/128	71/317	87.88	≤0.001*	65/81	58/32	22/15	9.567	0.008*
TG (mmol/L)	1.21 ± 0.56	1.23 ± 0.69	0.492	0.623	1.20 ± 0.52	1.24 ± 0.63	1.16 ± 0.56	0.327	0.721
TC (mmol/L)	4.05 ± 0.81	4.21 ± 0.87	2.403	0.017	4.07 ± 0.75	4.11 ± 0.90	3.82 ± 0.77	1.778	0.171
HDL (mmol/L)	1.54 ± 0.62	1.60 ± 0.56	1.224	0.222	1.49±	1.54±	1.52±	1.716	0.182
LDL (mmol/L)	2.21 ± 0.55	2.28 ± 0.63	1.443	0.149	2.23 ± 0.55	2.23 ± 0.57	2.06 ± 0.49	1.535	0.217
FBG (mmol/L)	4.43 ± 1.58	4.48 ± 1.36	0.389	0.697	4.39 ± 1.54	4.45 ± 1.53	4.58 ± 1.82	0.218	0.814
Abnormal Serum Lipid/Lipoprotein									
TG	33/240	56/332	0.756	0.226	16/130	11/79	6/31	0.770	0.680
TC	24/249	51/337	3.019	0.049*	11/135	11/79	2/35	2.138	0.350
HDL	19/254	18/370	1.633	0.135	11/135	6/84	2/35	0.224	0.894
LDL	10/263	25/363	2.470	0.080	5/141	5/85	0/37	2.344	0.310

Comparing the age, gender, FBG and Serum Lipid/Lipoprotein between subjects with smoking and non-smoking habit, and among groups with different smoking quantity, Using *χ*^2^ or Fisher’s exact test (where^a^ an expected cell count was < 5) for categorical variables and unpaired Student’s *t* test for continuous variables. In the testing, a *P* value < 0.05 was considered to be statistically significant. * *P* < 0.05 ** *P* < 0.01. FBG, fasting blood glucose; HDL, high density lipoprotein; LDL, low density lipoprotein; TC: Total cholesterol; TG: Triglyceride.

**Table 2:** Comparing the age, gender, FBG and serum lipid/lipoprotein between subjects with history of smoking and non-smoking, and between subjects with different smoking cessation (n = 661)

**Characteristics**	**With history of smoking**				**Smoking cessation**			
	**Yes n = 401**	**No n = 260**	***χ*2 or*t***	**P value**	**Yes n = 128**	**No n = 273**	***χ*2 or MD**	**P value**
Age (years)	93.41 ± 3.29	93.67 ± 3.49	0.980	0.327	93.50 ± 3.45	93.37 ± 3.22	93.62 ± 3.47	0.944
Gender ^a^								
(Male/Female)	178/223	38/222	63.120	≤0.001*	33/95	145/128	71/316	87.88
TG (mmol/L)	1.20 ± 0.63	1.26 ± 0.66	1.185	0.236	1.18 ± 0.75	1.21 ± 0.56	1.23 ± 0.69	0.492
TC (mmol/L)	4.11 ± 0.83	4.20 ± 0.88	1.333	0.183	4.24 ± 0.86	4.05 ± 0.81	4.21 ± 0.87	2.403
HDL (mmol/L)	1.55 ± 0.55	1.61 ± 0.64	1.301	0.191	1.57 ± 0.37	1.54 ± 0.62	1.60 ± 0.56	1.224
LDL (mmol/L)	2.24 ± 0.58	2.27 ± 0.63	0.663	0.508	2.21 ± 0.63	2.21 ± 0.55	2.28 ± 0.63	1.443
FBG (mmol/L)	4.49 ± 1.57	4.43 ± 1.25	0.483	0.630	4.59 ± 1.55	4.43 ± 1.58	4.48 ± 1.36	0.389
Hypertriglyceridemia	49/352	40/220	1.356	0.147	16/112	33/240	56/332	0.756
Hypercholesteremia	42/359	33/227	0.772	0.360	18/110	24/249	51/337	3.019
Lower HDL	25/376	12/248	0.782	0.240	6/122	19/254	18/370	1.633
Higher LDL	20/381	15/345	0.192	0.363	10/118	10/263	25/363	2.470

* *P* < 0.05 ** *P* < 0.01 vs. Using *χ*^2^ or Fisher’s exact test (where^a^ an expected cell count was < 5) for categorical variables and unpaired Student’s *t* test for continuous variables. In the testing, a *P* value < 0.05 was considered to be statistically significant. FBG, fasting blood glucose; HDL, high density lipoprotein; LDL, low density lipoprotein; TC: Total cholesterol; TG: Triglyceride.

### Current smokers had a lower TC serum level and a lower a prevalence of abnormal TC than nonsmokers, but no difference in the other serum lipid/lipoprotein levels and the prevalence abnormal of those

We examined the difference of serum Lipid/Lipoprotein and prevalence of abnormal serum Lipid/Lipoprotein between smokers and nonsmokers. The result showed that current smokers had a lower level of serum TC (4.05 ± 0.81 vs. 4.21 ± 0.87, *t* = 2.403, P = 0.017), and lower prevalence of abnormal TC(9.62% vs. 15.13%,*χ*^2^ = 3.018,P = 0.049) than nonsmokers. However, none of the difference in the serum TG, LDL and HDL levels was significant between current smokers and nonsmokers (1.21 ± 0.56 vs. 1.23 ± 0.69, *t* = 0.492, P = 0.623 for TG; 2.21 ± 0.55 vs. 2.28 ± 0.63, *t* = 1.443, P = 0.149 for LDL; 1.54 ± 0.62 vs. 1.60 ± 0.56, *t* = 1.224., P = 0.222 for HDL). None of the difference in prevalence of abnormal TG, LDL and HDL levels was significant between current smokers and nonsmokers (12.09% vs. 16.87%, *χ*^2^ = 0.756,P = 0.226 for TG; 3.80% vs. 6.88%, *χ*^2^ = 2.470,P = 0.080 for LDL; 7.48% vs. 4.86%, *χ*^2^ = 1.633,P = 0.135 for HDL). Current smokers showed no significantly higher risk for prevalence of abnormal Lipid/Lipoprotein. (See Tables 1 and 3).

**Table 3:** Effect of smoking and smoking cessationon abnormal serum lipid/lipoprotein in long-lived subjects

**Characteristics**	**Smoking**			**Smoking cessation**		
	**With/without (273/388)**	**OR**	**95% CI**	**With/without (273/128)**	**OR**	**95% CI**
TC						
NO. of Risk/Case	33/56			18/24		
Unadjusted		1.321	0.677–2.541		1.295	0.522–3.212
Adjusted		1.497	0.734–3.053		1.329	0.517–3.414
TG						
NO. of Risk/Case	24/51			16/33		
Unadjusted		1.126	0.693–1.830		0.876	0.442–1.738
Adjusted		1.074	0.639–1.083		0.895	0.438–1.827
LDL						
NO. of Risk/Case	10/25			6/19		
Unadjusted		1.310	0.503–3.459		1.804	0.519–6.267
Adjusted		0.957	0.343–2.670		1.564	0.430–5.685
HDL						
NO. of Risk/Case	19/18			10/10		
Unadjusted		0.671	0.341–1.313		0.709	0.274–1.832
Adjusted		0.732	0.355–1.508		0.753	0.283–2.003

(n = 661).

OR, odds ratio. Unadjusted: Wald Chi-square test with df = 1 was used; Adjusted multiple logistic regression was used to adjust for age, gender, FBG. FBG, fasting blood glucose; HDL, high density lipoprotein; LDL, low density lipoprotein; TC: Total cholesterol; TG: Triglyceride.

### None of difference in serum lipid/lipoprotein levels and the prevalence of abnormal those between subjects with and without smoking history, among groups with different smoking dosage, and between subjects with and without smoking cessation

We further examined the effect of smoking history (including current and former smokers), smoking dosage and smoking cessation on serum Lipid/Lipoprotein levels and the prevalence of abnormal those serum Lipid/Lipoprotein levels. The result showed none difference in serum Lipid/Lipoprotein (including TG, TC, LDL and LDL) and the prevalence of abnormal those (including TG, TC, LDL and LDL) was significant between individuals with and without smoking history, among different smoking dosage and between individuals with and without smoking cessation. There was also no significant difference in risk for the increased prevalence of abnormal those serum Lipid/Lipoprotein levels. (See Tables 2 and 3).

## Discussion

This study evaluated the effect of smoking habits (including current smoker, smoking history, smoking dosage and smoking cessation) on serum Lipid/Lipoprotein (including TG, TC, LDL and HDL). Current smoker had lower serum TC and lower risk for abnormal serum TC. The serum lower serum TC and lower risk for abnormal serum TC did not exist in individuals with smoking history, heavy smoking dosage and smoking cessation. Smoking habits did not have effect on serum TG, LDL and HDL.

In the present study, the result showed current smokers had lower level of TC and lower prevalence of abnormal TC [21-27]. This indicated that current smoking was associated with low serum TC levels, but none of difference in TC levels and prevalence of abnormal TC was significant difference among individuals with different smoking dosage. All of these results indicated that effect of smoking habits on serum TC was independent on smoking dosage.

Previous studies confirmed that in general population, current smoking increased TC levels and prevalence of abnormal TC, heavy smoking was associated with increased TC and higher risk for abnormal TC, and smoking cessation was associated with decreased TC and decreased risk for abnormal TC. Our result showed that the relationship between smoking habits and serum TC in long-lived subjects was different that in general population. This was an interesting finding. Mechanisms of difference could be explained by the following. In Chinese, mean life span was 77 years in 2011, in the present study, the all participants were all above 90 years. In the elderly, with aging, mortality rate increased. Smoking habits led to high TC in general adult. Smoking habits and hypercholesteremia were both definitive risk for cerebrovascular disease. Smoking habits and hypercholesteremia were also the confirmed risk for dementia, depression, disability and so on, all of these increased mortality rate in the elderly above 65 years [28,29]. The high mortality in the elderly could remove those with hypercholesteremia, which was caused by current smoking. This mean those with both habits of current smoking and hypercholesteremia were more possibly removed by the high mortality in the elderly, and left individuals with lower TC in current smokers. This led to a lower TC level in current smokers, compared non-smokers. This only a reasonable inference, which should be further confirmed using prospective cohort study.

To the best of our knowledge, this was the first research which focused on the association of smoking habits (including current smokers, with history of smokers, with different smoking dosage and smoking cessation) and serum Lipid/Lipoprotein among Nonagenarians/Centenarians. This study showed there was different in the association. Previous studies showed that current smoking and heavy dosage of smoking were both associated with increased TG, TC and LDL, and with decreased HDL. Smoking cessation could reverse the effect of smoking on serum Lipid/Lipoprotein [21-27]. The results of the present study were different that from those previous studies. In previous studies, the participants were not all long-lived subjects, but in the present, all participants were Nonagenarians/Centenarians. Longevity was multifactorial consequence, including genetic and environment factors. Genetic factors, which related with longevity, might include lipid metabolism gene, or include the gene, which could ant-disadvantage of smoking [28-30]. The genetic factor of longevity might have the function ant-disadvantage of smoking, including the effect of smoking on serum Lipid/Lipoprotein. This was also a reasonable inference, which should be further confirmed using experiment.

The present study showed that in long-lived subjects, smoking habits (current and former) were not associated with dyslipidemia, on the other hand, current smoker had lower TC and lower prevalence of abnormal TC. It had been confirmed that smoking habits (current and former) were definite risk factors of dyslipidemia. Therefore, from the results of the study, it could not conduct that in long-lived subjects smoking habits (current and former) were not risk factors for dyslipidemia, or that smoking habits (current and former) were prophylactic factors for dyslipidemia. As explanation above, longevial genetic factors included which might have the function ant-disadvantage of smoking, including the effect of smoking on serum Lipid/Lipoprotein. These genetic factors were still unknown, and it might be a field of research on Longevity.

Our study had some limitations that deserve a mention. First, 870 subjects aged 90 years or older volunteered for the PLAD Study, among these 870 volunteers, only 661 had non-missing data for the two main variables involved in the current analyses. There might be selection biases. Second, because of the cross-sectional nature of this study, we only concluded the association between smoking habits and serum Lipid/Lipoprotein. We could not conduct causal conclusions on it. Third, since this is a part of the PLAD, there might be a survival bias. However, this is inherent in a study of individuals of this age group. Forth, because of the cross-sectional nature of this study, the subjects might change their diets and the conditions related to elevated serum lipid concentrations. However, the lifestyles and food habits of the nonagenarians/centenarians were relatively stable and similar. Fifth, inflammation seems to be the mediator of most of the issues related to chronic disease. It would help provide some insight rather than just speculating. However, the data were all from a survey conducted in 2005, the data could not included inflammatory factor. Finally, there subjects with smoking habits in men had more than those in women. Gender might influence the association of smoking habits with serum Lipid/Lipoprotein. Data analyzed according to gender might remove the influence. There only 661 participants, we analyzed data according to gender, none of the serum Lipid/Lipoprotein among different smoking habits had significant different, so this caused the sample size too small and could not obtain a conclusion. Therefore, we reported results using the total sample, not according to gender.

In conclusion, among longevity Chinese, only current smoking was related with low TC levels. But the smoking habits were not associated with the other serum Lipid/Lipoprotein. The relationship between smoking habits and serum Lipid/Lipoprotein in long-lived subjects was different from that in general adults.

## Competing interests

The author(s) declare that they have no competing interests.

## Authors contribution

BR Dong provided the funds and the design of this study. YL Zhang drafted the manuscript , performed the study and offered the payment for publication. DQ Zhao carried out modifying the article and data analysis . CQ Huang participated in the laboratory test. All authors read and approved the final manuscript.
